# Targeting Type I Interferon Induction and Signaling: How Zika Virus Escapes from Host Innate Immunity

**DOI:** 10.7150/ijbs.83056

**Published:** 2023-06-04

**Authors:** Huan Hu, Yaxiu Feng, Ming-Liang He

**Affiliations:** 1 Department of Biomedical Sciences, City University of Hong Kong, Hong Kong, China.; 2 City University of Hong Kong Shenzhen Research Institute, Shenzhen 518057, China.

**Keywords:** Zika virus, type I interferons, interferons-stimulated genes

## Abstract

Zika virus (ZIKV) infection causes neurological disorders and draws great attention. ZIKV infection can elicit a wide range of immune response. Type I interferons (IFNs) as well as its signaling cascade play crucial role in innate immunity against ZIKV infection and in turn ZIKV can antagonize them. ZIKV genome are mainly recognized by Toll-like receptors 3 (TLR3), TLR7/8 and RIG-I-like receptor 1 (RIG-1), which induces the expression of Type I IFNs and interferon-stimulated genes (ISGs). ISGs exert antiviral activity at different stages of the ZIKV life cycle. On the other hand, ZIKV takes multiple strategies to antagonize the Type Ⅰ IFN induction and its signaling pathway to establish a pathogenic infection, especially by using the viral nonstructural (NS) proteins. Most of the NS proteins can directly interact with the factors in the pathways to escape the innate immunity. In addition, structural proteins also participate in the innate immune evasion and activation of antibody-binding of blood dendritic cell antigen 2 (BDCA2) or inflammasome also be used to enhance ZIKV replication. In this review, we summarize the recent findings about the interaction between ZIKV infection and type I IFNs pathways and suggest potential strategies for antiviral drug development.

## Introduction

Innate immune response is the first line of host defense against pathogens. And Interferons (IFNs) play an important role in innate immunity, including type I, type Ⅱ and type Ⅲ IFN 1-3. Type I IFNs, refer to a single subtype of IFN- β and multiple subtypes of IFN-α, have been found in all nucleated cells and proven to play a pivotal role in antiviral functions 4. Generally, the production of type I IFNs is induced by cell recognition of pathogen associated molecular patterns (PAMPs) 5. Type I IFNs bind to IFNs receptor (IFNAR), then activate receptor-associated janus kinases (JAK) and tyrosine-protein kinase (TYK), and subsequently lead to phosphorylation and activation of signal transducers and activators of transcription (STATs) 6, 7. The phosphorylated STAT1 (P-STAT1) or the phosphorylated STAT2 (P-STAT2) and the IFN-regulatory factor 9 (IRF9) form IFN-stimulated gene factor 3 (ISGF3), and the complex enters into the nucleus to trigger expression of IFNs-stimulated genes (ISGs) 8, 9. Some ISGs have been proven to exert diverse antiviral effects 10, 11. Meanwhile, several studies demonstrate that type I IFNs have effects on humoral response by upregulating antibody production or suppressing B‐cell linear epitopes 12, 13.

The induction of type I IFNs expression and type I IFNs signaling are key events of antiviral innate response 14, many members of Flavivirus genus have been proven to interact with type I IFN response. Flavivirus genus consists of more than 70 RNA viruses, including Dengue virus (DENV), West Nile virus (WNV), Zika virus (ZIKV), and Japanese encephalitis virus (JEV) etc. Flavivirus genus can cause health damage to varying degrees. DENV is associated with dengue shock syndrome / dengue hemorrhagic fever and ZIKV is related with microcephaly and so on. Type I IFNs is important to resist flavivirus infection. Studies have demonstrated that IFNAR1 (Interferon Alpha and Beta Receptor Subunit 1) deficient mice were susceptible to DENV and ZIKV infection 15. On the contrary, almost every member of the flavivirus genus can evade the type I IFN response. For example, multiple non-structural proteins (NS) of DENV have been shown to inhibit type I IFNs and the downstream signaling pathway. DENV NS4A blocks the interaction between RIG-I and MAVS 16. DENV NS2A and NS4B suppresses TBK1 phosphorylation 17. And WNV NS proteins are responsible for the degradation of IFNAR 18. All of them were well summarized in previous reviews.

ZIKV, as an emerging pathogen, was first discovered in the Zika forest of Uganda in Africa in 1947 19. ZIKV can be sexually and vertically transmitted 20-22, and the bite by the infected female Aedes mosquitoes is the most common route of transmission 19. Clinical symptoms of people infected with ZIKV are mostly described as asymptomatic, while 20%-25% of the infected will develop self-limiting flu-like symptoms after a duration of 4-10 days 23. An increased risk of neurologic complications associated with ZIKV infection has been observed, such as Guillain-Barré syndrome, microcephaly and so forth 24-26. The World Health Organization (WHO) declared ZIKV infection as a Public Health Emergency of International Concern in 2016 27.

ZIKV has a single-stranded RNA genome with positive sense, encoding three structural and seven NS proteins 28. The envelope (E) protein and membrane (M) protein form an icosahedral shell anchored in a lipid membrane 29. The E protein binds to cell receptor that mediates viral attachment and membrane fusion 30. The E protein is also an important target for immune recognition. NS proteins of ZIKV include NS1, NS2 (NS2A, NS2B), NS3, NS4 (NS4A, NS4B), NS5. Most of them are correlated with viral replication and immune response evasion 30 (Table 1).

This review focuses on the interaction between ZIKV infection and Type I IFN signaling, which would help us to identify potential strategies for antiviral drug development.

## Pattern recognition receptors (PRRs) associated with ZIKV infection

The recognition of ZIKV by PRRs is fundamental in producing type I IFNs. The viral replication intermediates of double-stranded RNA (dsRNA), RNA transcripts and protein are activator for PRRs 52, 53. It has been observed that the expression of several PRRs involved in the innate immune response in ZIKV-infected astrocytes, such as membrane-anchored Toll-like receptors (TLRs), RIG-I-like receptors (RLRs) 54. Inhibition of PRRs signaling in testicular germ cells (TGCs) leads to the prolonged replication of ZIKV, and such phenomenon can be reversed by exogenous IFNβ 55.

### TLRs

**Figure 1:**
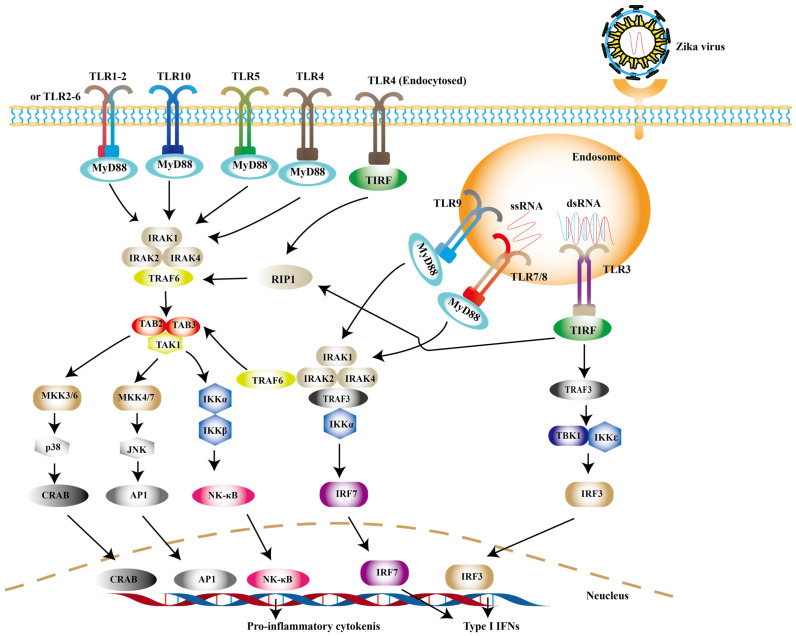
** TLR signaling pathways.** Upon ZIKV infection, TLR3 and TLR7/8 are mainly activated to induce the expression of type I IFNs. And other TLR family members can be activated during other viral infection.

TLRs are widely distributed on the cell surface or in endosomal membranes of effector cells. TLR signaling pathways are crucial pathways in innate immune defense. Up to now, 10 human TLRs have been found in humans and the function of TLR1-9 has been confirmed. TLR-3, -7, -8 and -9, which locate in the endosome, are the key players involved in antiviral immunity 56. After PAMPs binding to PRRs, the activation of downstream signaling depends on myeloid differentiation primary response 88 (MyD88) or toll-interleukin 1 receptor domain-containing adapter (TRIF) induces type I IFNs 57. Among them, TRIF is required by TLR3 or TLR4, while the rest recruit MyD88 58. Experimental observations indicate that TLR7/8 agonist R848 is an inhibitor for blocking ZIKV replication in monocytes 59. TLR7/8 are sensors for single-stranded RNA (ssRNA) 60. The connection of MyD88 and TLR7/8 leads to the phosphorylation of interleukin-1 receptor-associated kinases (IRAKs), followed by the activation of TRAF6, TRAF3, IKKα and IRF7, resulting in the release of type I IFNs 61 (Fig. 1).

It is noteworthy to mention that TLR3 may exhibit opposite effects in different cell types. On one hand, TLR3 can recognize the intermediate of double-stranded RNA (dsRNA), and then recruits TRIF to phosphorylate interferon regulatory factor 3 (IRF3), nuclear factor-κB (NF-κB) successively, which leads to the production of type I IFNs ultimately 62 (Fig. 1). On the other hand, it may be related with pathogenicity of ZIKV. TLR3 is highly expressed in the early development of the brain 63. The activation of TLR3 leads to dysregulation of neurogenesis in neural progenitor cells (NPCs) and apoptosis, the possible cause of microcephaly in newborn baby 64. In primary human astrocytes, TLR3 contributes to ZIKV-associated neurodevelopmental disorders by releasing inflammatory factors 65. In addition, TLR3 enhances ZIKV replication by suppressing other IFNs production and their signaling 66. Such detrimental effect of TLR3 can also be observed in WNV, another virus of the genus Flavivirus. Studies have revealed that TLR3-dependent inflammatory response caused by WNV leads to neuronal injury 67.

### RLRs

RLRs are cytoplasmic viral RNA sensors, including retinoic acid-inducible gene I (RIG-I), melanoma differentiation-associated gene 5 (MDA5) and laboratory of genetics and physiology 2 (LGP2) 68. The short double stranded RNA with 5'- triphosphate (3P) terminal 69 and viral double-stranded RNAs (dsRNA) 70 are ideal ligands for RLRs, while the 5′ region of ZIKV's genome is such a ligand 71, 72. In ZIKV- infected cells, RIG-1-mitochondrial antiviral signaling (MAVS) signaling pathway is a major pathway against ZIKV, especially in the central nervous system (CNS) 73. RIG-I and MDA5 are mainly distributed in astrocytes and microglia 74. Neural stem cells (NSCs) infected with ZIKV can activate RIG-I pathway to induce the expression of IFN-β, which limits the transmission of ZIKV 74. And RIG-I-mediated pathway effectively protects human dermal fibroblasts and epidermal keratinocytes against ZIKV infection 73.

**Figure 2:**
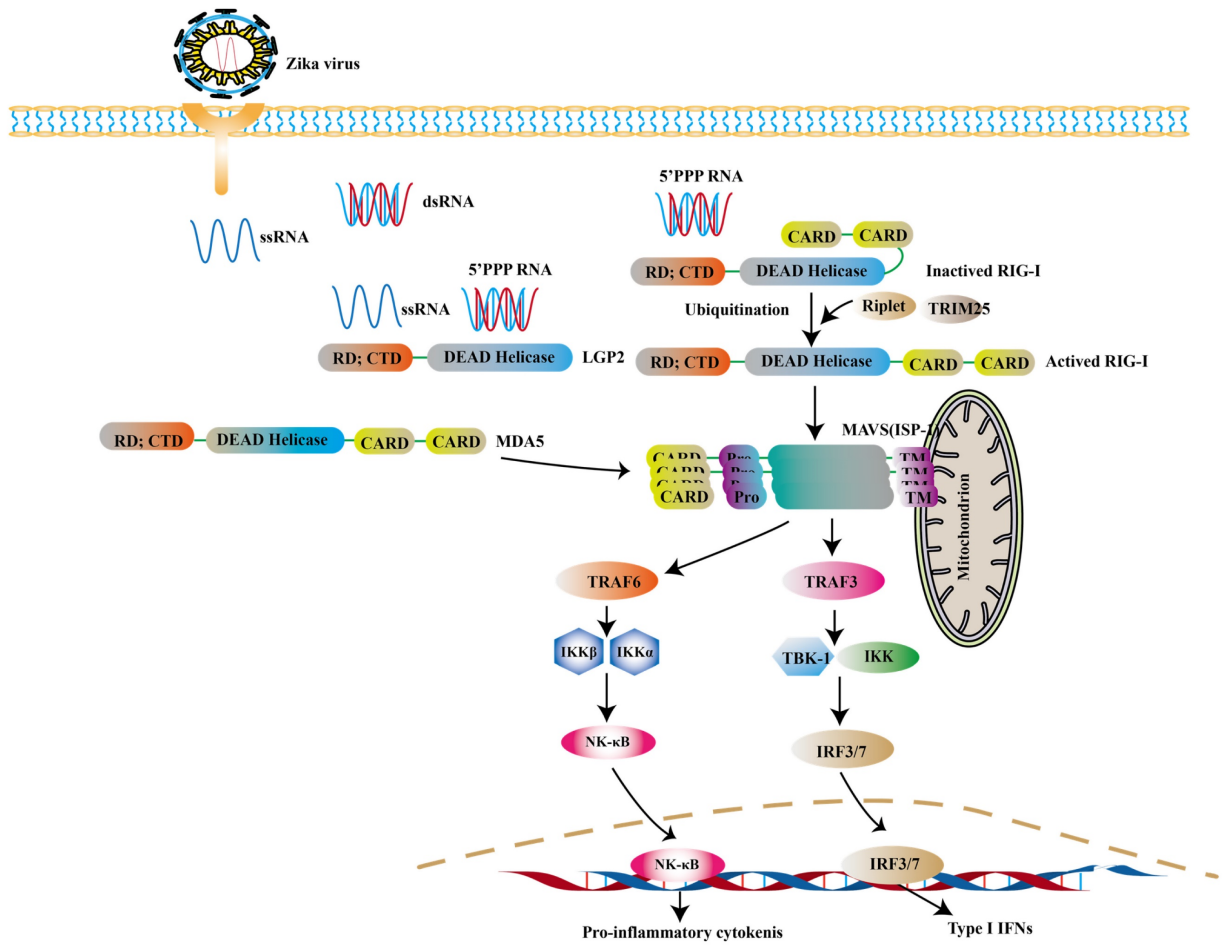
** RIG-I is mainly activated during ZIKV infection.** The 5'ppp RNA of ZIKV activates the RIG-I with the help of Riplet and TRIM25. And the activated RIG-I translocates to mitochondria to interact with MAVS. And then the TRAF3 is phosphorylated to induce the expression of type I IFNs. Apart from this, the activation of MDA5 can be observed in other viral infections.

RIG-I and MDA5 are all comprised of two N-terminal caspase activation and recruitment domains (CARD), a central DExD/H box RNA helicase domain that has the ability of hydrolyzing ATP and binding or possibly unwinding RNA, and a C-terminal repressor domain (RD) embedded within the C-terminal domain (CTD) 75, 76. After CTD binding with intracellular virus-derived, CARD is exposed, resulting in the activation of RIG-I. And then the polyubiquitination of RIG-I is triggered through two ubiquitin E3 ligases, tripartite motif-containing 25 (TRIM25) and Riplet 77. The interaction between polyubiquitinated RIG-I and MAVS leads to further recruiting a group of molecules to activate TANK binding kinase 1 (TBK1) - IκB kinase (IKK) complex. These kinases then activate transcription factors such as IRF3, IRF7, thereby inducing the expression of genes encoding type I IFNs and the production of pro-inflammatory cytokines 78-80 (Fig. 2).

## The antiviral ability of Interferon-stimulated genes (ISGs)

**Figure 3:**
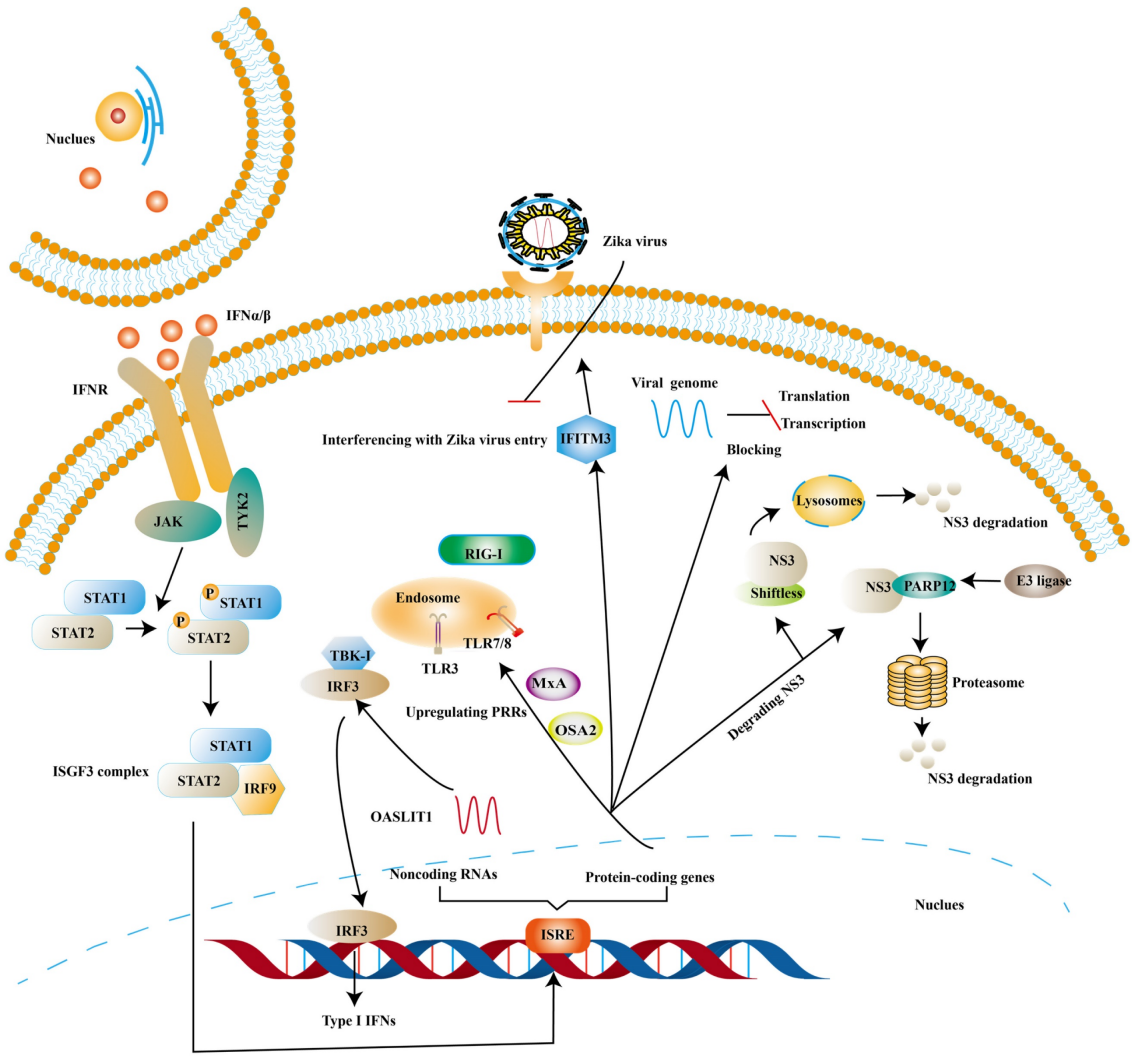
** ISGs elicit the antiviral state against ZIKV.** The binding of Type I IFNs and IFNAR activate classical JAK/TYK2 pathway, which leads to the formation of ISGF3. And then ISGF3 translocates into the nucleus to induce the expression of ISGs. Finally, various species of ISGs exert antiviral effects at different steps of the ZIKV life cycle or strengthens the expression of Type I IFNs.

ISGs, induced by the binding of IFNs and IFNAR, have been proven to inhibit viral infection at different stages of the viral replication cycle 81, 82. One of the canonical activation pathways to induce the transcription of ISGs is JAK-STATs pathway (Fig. 3) 83. ISGs stimulated by type I IFNs comprise protein-coding genes and noncoding RNAs (ncRNAs). At present, protein-coding genes are more than 300 and ncRNAs can be divided into short ncRNAs and long ncRNAs based on the length 84, 85.

Some ISGs exert their antiviral effects by positive feedback on the induction of type I IFNs though ISGs are the effector molecules of type I IFNs response. OASL-IT1, a type of ncRNAs, can trigger production of IFN-β by regulating IRF3 and NF-κB positively to help epithelial cells resist ZIKV infection 85. The same trend is mirrored in ISGs encoding antiviral protein. Myxovirus resistance protein A (MxA) is found to strengthen the expression of type I IFNs and activate JAK-STATs signaling pathway by upregulating the expression levels of PRRs 85. *In vitro* experiments suggest that antigen processing type 1 (TAP1) inhibits ZIKV infection by means of phosphorylating TBK1 and IRF3 86. 2′, 5′-oligoadenylate synthetase (OAS) 2 can also exert its antiviral effects by means of enhancing the expression of type I IFNs 87. The possible mechanism is OAS / RNase L pathway, in which OAS activates RNase L (a latent endoribonuclease) and the activated RNase L cleaves both host and viral RNA indiscriminately. In turn, the cleaved RNA can stimulate PRRs to reinforce the production of type I IFNs 88.

**Figure 4:**
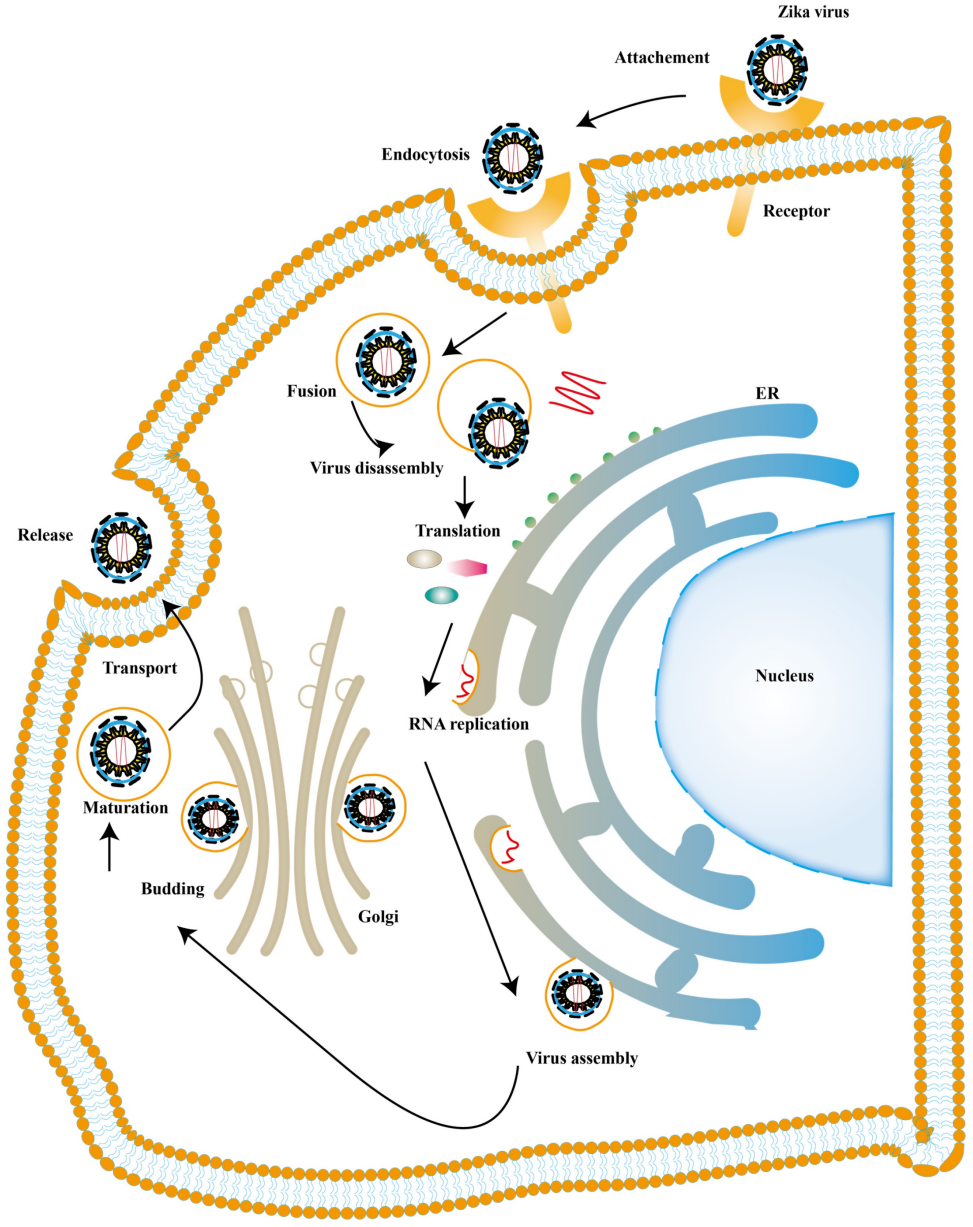
** ZIKV life cycle.** The ZIKV infection cycle can be divided into 7 stages, including viral attachment, membrane fusion, endocytosis, transcription and translation, genome replication, virion assembly, maturation and release.

There is also a significant part of ISGs exert antiviral activity at multiple steps in the ZIKV replication cycles. The ZIKV life cycle starts with the E protein binding to cell surface receptor and ZIKV enters the cell through endocytosis. The low pH in endosome induces conformational rearrangement of E protein, which leads to the release of the genome. The viral genome is translated into a polyprotein with the help of the host translation system and it is finally cleaved into three structural and seven NS proteins. Subsequently, the viral replication takes place within vesicles. Viral RNA is packaged in the endoplasmic reticulum (ER) to an immature virion and then it is transferred into golgi vesicles to form mature virions. The mature virion can be released to the extracellular space 89 (Fig. 4). Experimental data shows that small membrane-associated interferon-inducible transmembrane proteins (IFITMs) 3 suppresses ZIKV infection by inhibiting cytosolic entry of ZIKV or its early transcription 90 and the possible mechanism is that it blocks fusion pore formation and inhibits ZIKV viral genome and proteins entry into the cytosol after ZIKV-host binding 91, 92.

In addition to this, ISGs can inhibit viral ZIKV replication by degrading NS3. NS3 possesses helicase and RNA triphosphatase activities, playing an essential role in virus replication 93. Ubiquitin-proteasome system and lysosomal proteolysis are two main intracellular protein degradation pathways. Theoretically, ubiquitin system refers to the ubiquitinated protein being degraded by the proteasome. And the ubiquitination of protein is completed by a cascade of reactions, including ubiquitin-activating enzyme E1, ubiquitin-conjugating enzymes E2 and ubiquitin ligase E3 94. Lysosomal proteolysis is that protein is delivered into lysosomes and degraded by a series of proteases 95. PARP12 belongs to the family of poly-adenosine 5′-diphosphate-ribose (PARPs), which consists of PARP domain, four zinc-finger (ZnF) domains and WWE domain (named after three of its conserved residues, including two conserved tryptophan (W) residues and a glutamic acid (E) residue). NS3, bound by PARP domain, is ubiquitylated by the E3 ligase and degraded by the proteasome 96. NS1 can also be degraded by such a pathway 96. Further analyses indicate that PARP11 and PARP12 seem to have a synergistic effect in the defense of ZIKV 97. Viperin, a member of the radical S-adenosyl methionine (SAM) superfamily of enzymes, has been proven to degrade NS3 via proteasome-dependent manner 98 and Lys358 of NS3 is an essential amino-acid for viperin against ZIKV 99. Shiftless (also known as C19orf66) is reported to degrade NS3 by lysosomal proteolysis. Shiftless is a conserved ISG in mammals, can bind NS3 protease domain and then NS3-shiftless is localized in lysosomes to promote the degradation of NS3 100. However, according to Natasha et al. shiftless can bind the ZIKV RNA to inhibit viral replication 101.

**Figure 5:**
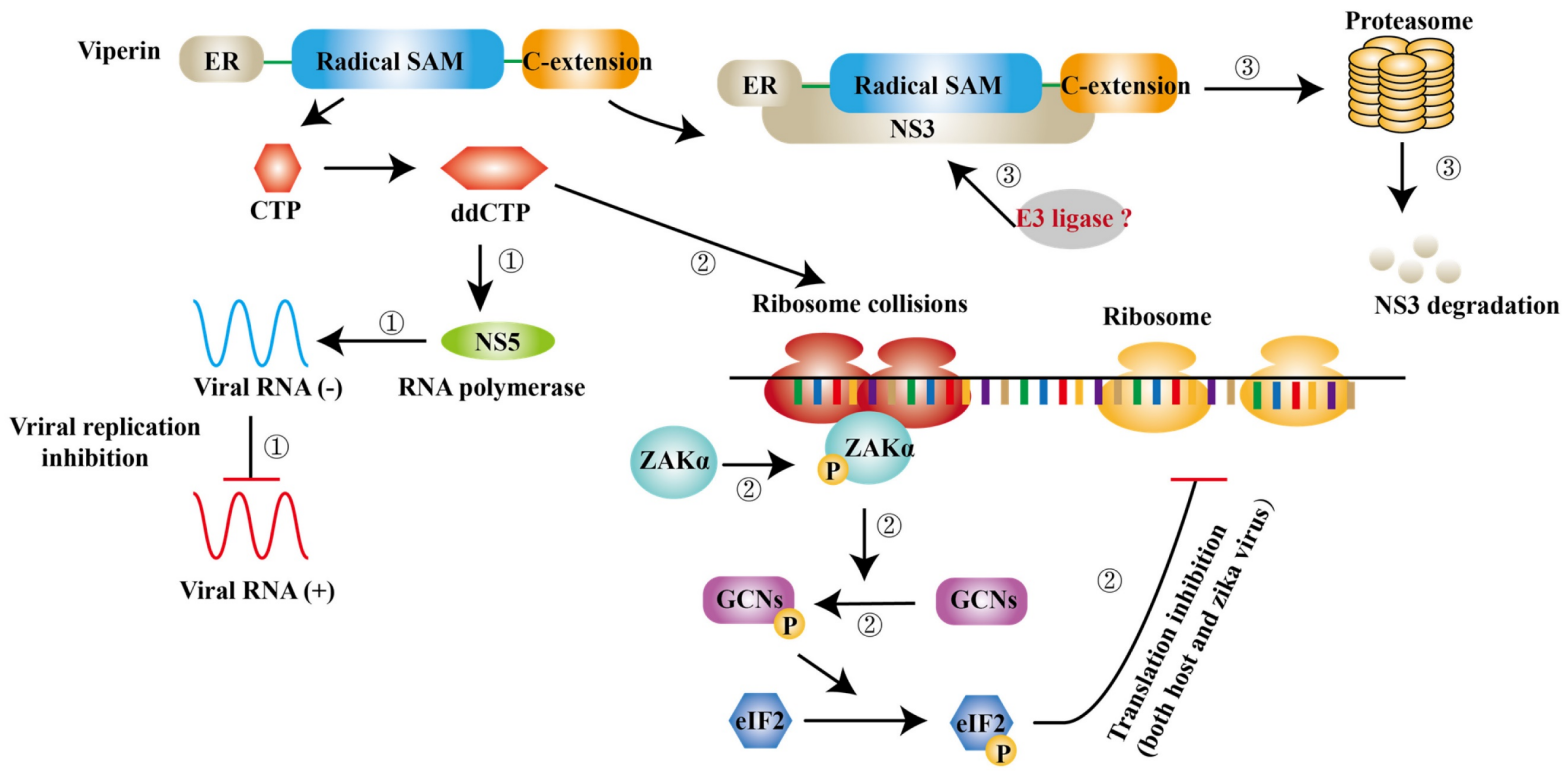
** Viperin inhibits ZIKV infection via different mechanisms.** ① ddCTP can be a chain terminator for RNA polymerase and then inhibit viral replication. ② ddCTP is able to trigger ribosome collisions and activates ZAKα and induces the phosphorylation of GCNs and eIF2 successively. As a result, translation of both host cells and ZIKV is inhibited. ③ The complex of NS3 and viperin initiates NS3 degradation via a ubiquitin proteasome pathway.

ISGs have also been reported to inhibit viral transcription and translation. Apart from degrading NS3, viperin is found to block the minus-strand RNA or plus-strand RNA synthesis to limit viral protein expression 59. Jack et al. further suggest that viperin restricts the translation of ZIKV genome via triggering ribosome collisions pathway, and it even restricts the translation of other genomes in cells 102. ddhCTP is the enzymatic product of viperin, which can activate the GCN2, an eIF2α kinase and the activated eIF2α blocks translation initiation to restrict protein expression immediately after 103, 104. ISGs can also impair viral RNA to prevent viral infection. ISG20 is a 3′-5′ exonuclease and can degrade ZIKV RNA to block viral replication in cytrophoblast cells of first‐trimester placenta 105 (Fig. 5).

Type I IFNs and type I IFNs- mediated ISGs empower host antiviral ability, however, they may involve in the pathogenesis of ZIKV. Type I IFNs induced by ZIKV interferes with the development of placental labyrinthine zone in mice, finally resulting in fetal (mouse) death and the exact mechanism still needs to be explored 106.

## Antagonism of innate immunity by ZIKV

**Figure 6:**
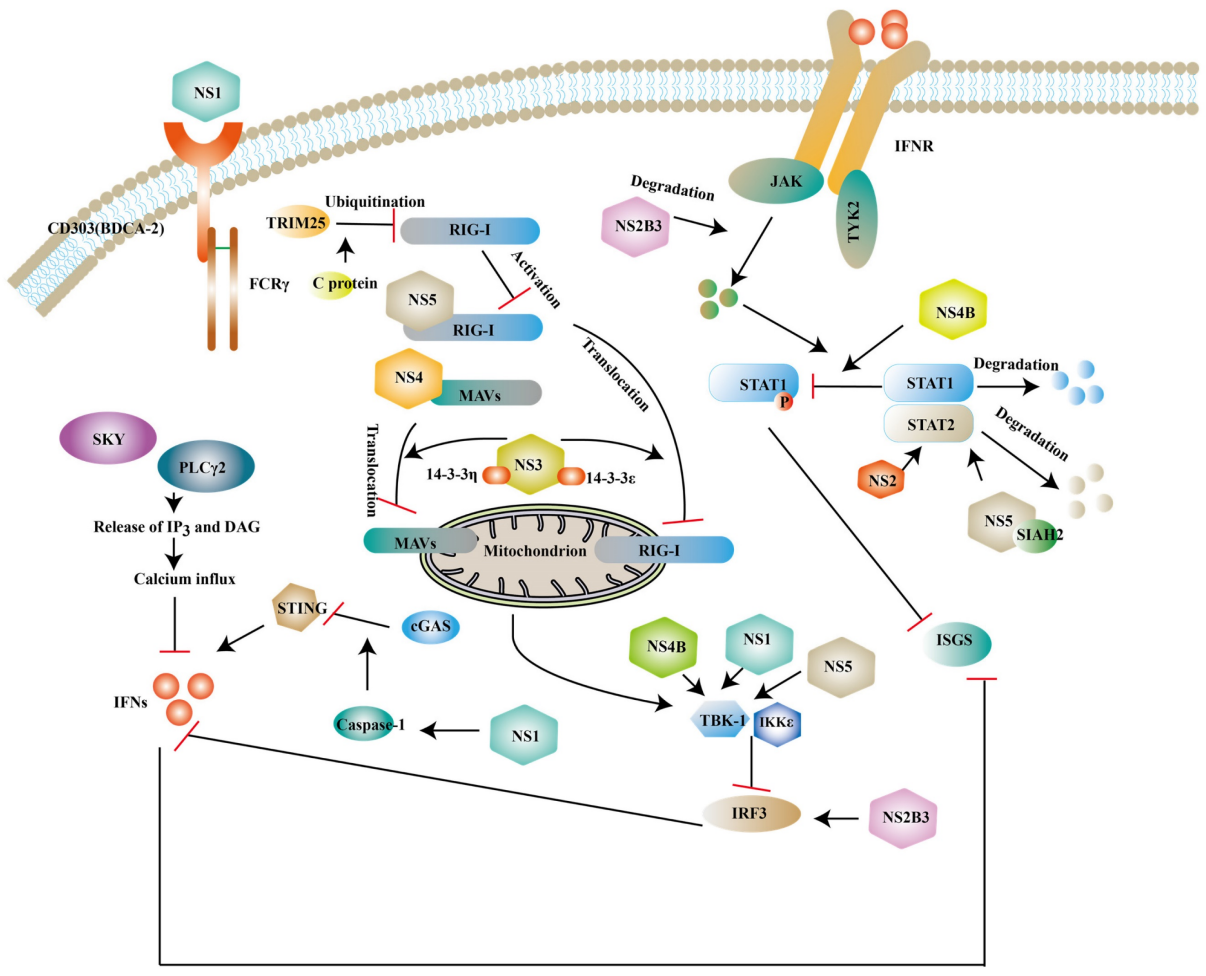
** Structural and NS proteins of ZIKV antagonize the host innate immune response.** NS2B3, NS3, NS4B, NS5 can block the expression of type I IFNs and ISGs via inhibit phosphorylation of some factors. NS2B3 also can degrade JAK. NS1 can interact with TBK1 or enhance active caspase-1 stability to inhibit the expression of type I IFNs. While NS5 and NS4 can also prevent RIG-I/ MAVs translocation.

Although innate immunity is an important immune response against ZIKV infection, studies have shown that ZIKV can escape innate immunity by different ways. Human dendritic cells have limited immunogenicity after ZIKV infection, partly due to the viral antagonism of type I IFN response. For example, IFN- β upregulates the expression of major histocompatibility complex class I (MHC-I) and inhibits the killing effect of natural killer cells against ZIKV 107 and NS proteins of ZIKV inhibit the expression of type I IFNs or its downstream molecules to antagonize the innate immunity of host 108, 109 (Fig. 6).

### NS1

NS1 is a glycoprotein and it can be secreted into the extracellular space in the form of a hexameric lipoprotein particle (sNS1) 110. BDCA2 (also known as CD303) is a C-type lectin and the activation of it leads to a reduction of type I IFNs, which presumably be related to calcium mobilization and PLCγ2 phosphorylation 111, 112. One study has revealed that NS1 can activate BDCA2 to limit type I IFNs production based on its N-glycosylation sites 113. In the CNS, NS1 can upregulate the miR-146a expression and followed a decrease of TRAF6 in human microglial cells, which leads to the reduction of type Ⅰ IFNs 114. NS1 also interacts with TBK1 to inhibit the expression of IFNs 115. Specifically, the NS1 A188V mutation leads to less phosphorylation of TBK1 116. In addition to interfering with the typical type I IFNs activated pathway, NS1 is also reported to weaken the expression of type I IFNs via activating inflammasome. Caspase-1 is a protease and it mediates the cleavage of cyclic GMP-AMP synthase (cGAS) 117. cGAS is a DNA sensor located in the cytosolic and cells without it are more vulnerable to some flavivirus according to John et.al 118. ZIKV can enhance active caspase-1 stability via activating the nucleotide-binding domain and leucine-rich repeat protein-3 (NLRP3) inflammasome or lowering the caspase-1 degradation, which leads to the cleavage of cGAS 119.

### NS2/NS3

As revealed by recent studies, NS2A impairs the activation of the NF-κB promoter and the exact mechanism needs further exploration 120. NS2A consists of 226 amino acids and NS2A_51-100_ localized in the ER has been proven to mediate the degradation of STAT1 and STAT2, but it is still unclear that such a degradation occurs in proteasomes or lysosomes 121. Structural study has shown that NS3 protease domain is folded as chymotrypsin-like and it is enwrapped by NS2B polypeptide 122. NS3 can serve as a protease with the help of NS2B, which is responsible for polyprotein processing and maturation of structural/NS proteins 40. Mediator of IRF3 activation (MITA, also known as STING or ERIS) is a scaffold protein located in the ER and it can recruit TBK1 or IRF3 to MAVS to transmit signals to downstream molecules 123. The interaction of NS2B3 with the MITA leads to the degradation of MITA via the ubiquitin proteasome pathway 124. And the overexpression of NS2B3 can degrade JAK protein levels in a proteasome-dependent manner to impair downstream signaling pathway 115. Apart from the above, NS3 has been reported to interrupt the translocation of RIG-I and MDA5 from the cytosol to the mitochondria via interacting with 14-3-3ε protein and 14-3-3η protein and such a translocation is required for the activation of TRAF3 125.

### NS4

NS4A is divided into a water-soluble N-terminal cytoplasmic domain and three predicted transmembrane (pTMs) segments. It has been demonstrated that NS4A binds to N-terminal CARD of MAVS to inhibit the interaction of RIG-I and MAVS and which part of NS4 mediates this binding needs further investigation 126. NS4A also plays an antagonistic role in the production of NF-κB 120. NS4B can reduce TBK1 activation. One possible mechanism is that NS4B promotes Cholesterol metabolic enzyme 7-dehydrocholesterol reductase (DHCR7) expression. DHCR7 is the inhibitor of TBK1 and IRF3 activation 127. *In vitro* studies have shown that NS4B can strongly inhibit the phosphorylation of STAT1 without affecting the total expression of STAT1 46. Some studies have showed that NS4B and NS1 protein have synergistic effects to inhibit type I IFN response. IFNβ degrades NS2B-NS3 by autophagic degradation. Wu et al suggested that NS1 or NS4B impaired NS2B3 degradation to attenuate type I IFN response and the detailed mechanisms needs to be explored 115.

### NS5

NS5 is composed of an N-terminal MTase and a C-terminal RdRp domain, plays an important role in viral replication and suppresses the RIG-I/MAVs pathway at different levels. The interaction of polyubiquitinated RIG-I and MAVS is necessary for activating downstream signaling pathways. NS5 is proven to impair the polyubiquitination of RIG-I 128. Some studies suggest that NS5 suppresses the activation of TBK1 129 or interacts with IKKε to decrease IRF3 phosphorylation 130. Recently, it has been shown that NS5 interacts with IRF3 localized in the nucleus to inhibit the transcription of type I IFNs during ZIKV infection 131. Another important target of NS5 is STAT2, which consists of an N-terminal domain (ND), a coiled coil domain (CCD), a DNA binding domain, a linker domain (LD), a SH2 domain, and a transcriptional activation domain (TAD) 132. It has been demonstrated that the expression of NS5 degrades CCD of STAT2 via the proteasome pathway 132, 133, which may be related to the MTase domain of NS5 134. A computer simulation indicates that seven in absentia homolog (SIAH) 2, an E3 ubiquitin ligase that mediates ubiquitination and proteasomal degradation, can be recruited by NS5 to degrade STAT2, which needs to be experimentally validated 135. However, Jun Shu et. al suggest that NS5 only slightly degrades STAT2 and it is the restriction of ZIKV on host de novo protein synthesis that accelerates the degradation of STAT2 136. Certain members of the genus Flavivirus are also proven to repress host protein synthesis 137. And it is unclear whether the suppression of host protein synthesis of other Flavivirus accelerates STAT2 degradation. Additionally, a study demonstrated that small-ubiquitin-like modifier (SUMO) NS5 of ZIKV can form discrete punctate nuclear bodies (NBs) with STAT2 and thereby remove promyelocytic leukemia (PML) protein from NBs. STAT2/NS5 NBs repress ISG transcription 138.

### Structural proteins

**Figure 7:**
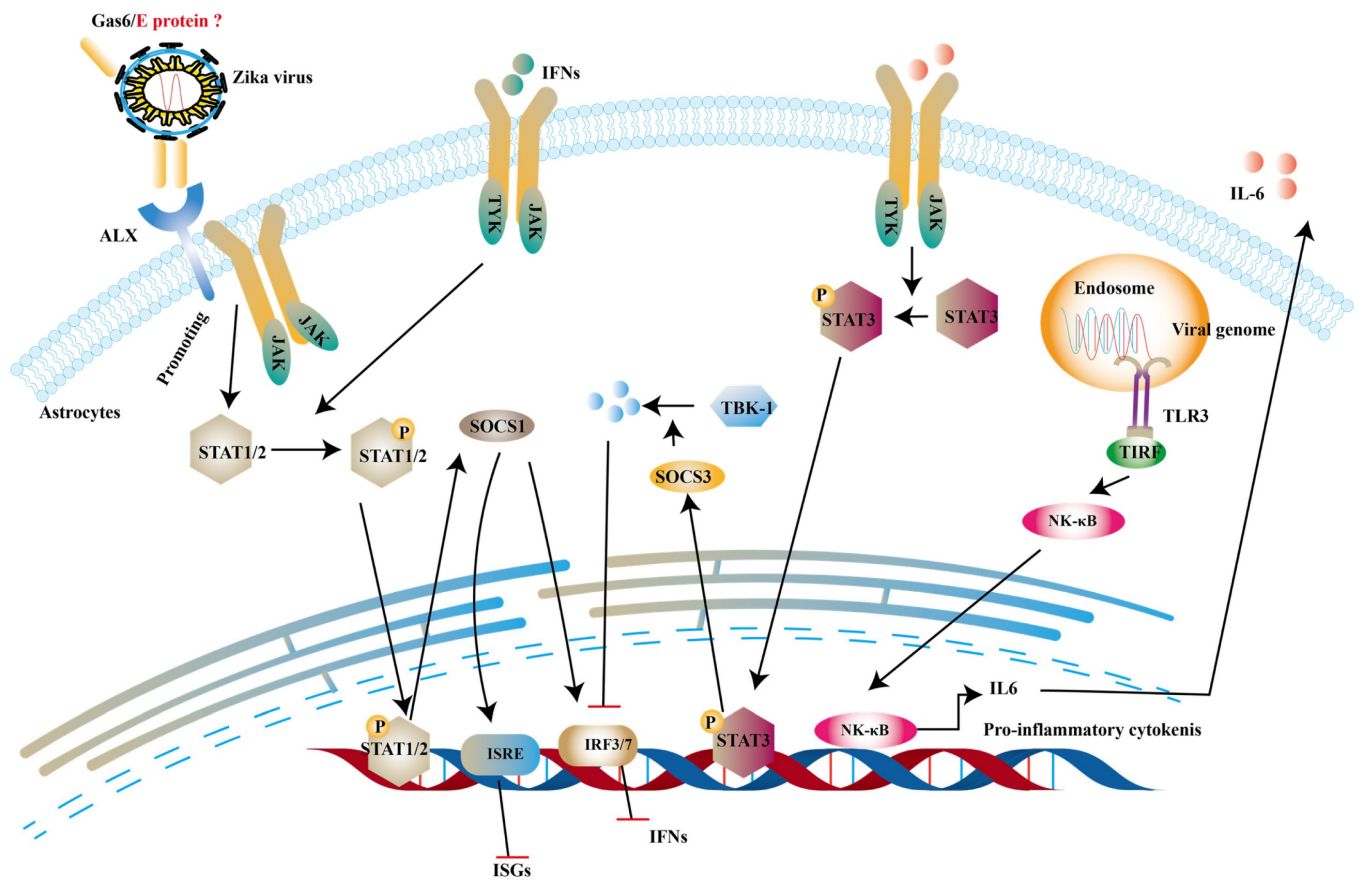
** ZIKV inhibits type 1 IFNs pathway via IL-6 and ALX receptor.** The dsRNA of ZIKV can activate TLR3 to induce the expression of IL-6 and in turn IL-6 phosphorylates STAT3 to trigger the production of SOCS3 to degrade TKB-1. On the other hand, the complex of Gas6 and E protein can promote the phosphorylation of STATs, which induce the expression of SOCS1. And it can inhibit the production of IFNs and ISGs.

Structural proteins are necessary components of ZIKV virions along with the nucleic acid. And they are also involved in innate immune evasion. Tripartite motif protein 25 (TRIM25) is a type of ubiquitin ligase E3 and mediates polyubiquitination of RIG-I, thereby activating downstream signal transduction 139. One study reported that capsid protein of ZIKV can bind to TRIM25 to prevent ubiquitination RIG-I 140. TAM receptor tyrosine kinases include Tyro3, Axl, Mer and Axl are important co-factors in ZIKV entry into human fetal endothelial cells 141. It is stated that growth arrest specific gene 6 (Gas6), a ligand of Axl, firstly binds to phosphatidylserine on the E protein of ZIKV and subsequently helps ZIKV to bind to TAM 142. Chen et al. indicates that the activation of TAM leads to the production of SOCS1, which inhibits type I IFNs and ISGs 143. SOCS1 belongs to SOCS proteins and negatively regulate JAK-STATs pathway to restrict the expression of ISGs 144. In addition to the this, interleukin-6 (IL-6) promotes production of SOCS3. And then SOCS3 suppresses the levels of type I IFNs and ISGs through degrading TBK1 145 and decreasing STAT1 phosphorylation 146 (Fig. 7).

## Subgenomic flavivirus RNAs and the genomic RNA of ZIKV

Subgenomic flavivirus RNAs (sfRNAs) are products of incomplete degradation of viral genome. They have been proven to be involved in antagonizing type I IFNs responses 147, 148. Two species of sfRNAs are produced during ZIKA infection, as a consequence of stalling of host 5′- 3′ exoribonucleases in the 3′ untranslated region (UTR) of ZIKV genome 149, 150. It has been reported that ZIKV sfRNAs inhibited type 1 IFN response, as evidence by that ZIKV-derived sfRNA suppressed type I response at the cellular level 150, 151. Some evidence suggests that ZIKV sfRNAs and ZIKV NS5 act in cooperation to inhibit STAT1 phosphorylation 152.

The genomic RNA (gRNA) can be chemical modificated during flavivirus infection and RNA methylation modification is the most common. ZIKV RNA methylation occurs at different sites. For example, the ribose 2′-oxygen (2′-O), the position N-6 of adenosine, and the nitrogen on position 7 (N-7) of guanosine 153. Some studies indicate that 2′-O-methylation of the cap structure can escape the recognization of PRRs 154, 155. Experimental evidence shows that 2′-O methylation of WNV can evade the ISG, tetra-tricopeptide repeats (IFIT) response 156 and DENV deficient 2′-O methylation mutant was more sensitive to IFN 157.

## Conclusions and future perspectives

Currently, there are no licensed vaccines or approved drugs to prevent or treat ZIKV infection. Drug targets can be either host proteins or viral proteins. Targeting host factors may trigger immune antiviral responses or disrupt the viral life cycle, while targeting viral protein could directly damage or suppress the virus life cycle 158.

Type I IFNs plays an important role in defending against viral infection and type 1 IFNs system has been used as a host-targeting antiviral method. However, the interaction between ZIKV and type 1 IFNs system is complicated. Better understanding their interactions would help us to identify potential molecular targets for treating ZIKV infection. As mentioned in this article, ZIKV has different mechanisms to evade the innate immunity, especially the inhibition of the type I IFN response. At present, many research studies focus on ZIKV proteins directly act on the key factors involved in type I IFNs induction pathway and type I IFNs signaling pathway. A NS protein can interfere with multiple factors involved in the typical type I IFNs system and there are cascade reactions between those factors, such as TBK1 and IRF3. The inhibitory mechanism of NS proteins on these interrelated factors needs further exploration. NS proteins are usually composed of several domains and some of the inhibitory effects of NS proteins on type I IFNs are not localized to specific domains or sites. This allows us to conduct detailed studies about this aspect in the future. And some proteins induced by ZIKV can also indirectly inhibit the expression of type I IFNs and ISGs, such as SOCS proteins and this field needs further investigation. A recent study showed that ZIKV infection stimulates Pim1 kinase expression, which serves as a negative regulator of type I IFN signaling. The Pim1 inhibitors potently inhibited ZIKV reproduction 159, suggest a strategy for developing anti-ZIKV drugs although the underlying mechanism is to be further investigated.

Type I IFNs therapy has been considered an effective antiviral therapy, for example, IFN-β treatment can repress the transcription of ZIKV in primary human vaginal and cervical epithelial cells 160. Apart from this, the combination of type 1 IFNs and drugs has evident limitations in viral replication. Sofosbuvir 161, ribavirin 162 and bromocriptine 163 combined with IFN-α/β have also been proved to protect against ZIKV infection *in vitro*. In addition to that, antiviral proteins encoded by ISGs may applied in protecting against ZIKV infection and some drugs with positive feedback on the type I IFNs induction pathway and type I IFNs signaling pathway are considered. After all, there are some disadvantages in using type I IFNs as drug therapy, such as short half-life *in vivo*, high cost, unexpected side effects and high dosage 164. In addition to this, some researchers think it is an effective vaccine strategy to modify the NS proteins sites. ZIKV NS4B_C100S_ mutant has been reported to induce higher type 1 IFNs and it enhances CD4^+^ and CD8^+^ T-cells responses in immunized mice 165. Whether the same effect is achieved by modifying the NS proteins sites involved in innate immune evasion should be investigated in the future study.

## Figures and Tables

**Table 1:** Structure and functions of ZIKV proteins

Name	Functions	Functional domains
E	Viral entry, membrane fusion and eliciting neutralizing antibodies 31	Domain I, domain II and domain III 29
M	Assisting E protein folding and preventing ZIKV premature fusion 31	A loop at the N terminus (M-loop), a stem and a transmembrane region 29
C	Facilitating transfer of viral genome into the host cell, recruitment viral genome and genome encapsulation 32	Four α helices with a long pre-α1 loop 33
NS1	Viral replication, antigenic marker, immune evasion, pathogenesis and inducing a specific immune response 34, 35	A β-hairpin domain, a wing domain and a β-ladder domain 36
NS2A	Viral replication, viral assembly or secretion and immune evasion 37-39	A membrane-traversing segment and six segments associated peripherally with the ER membrane 37
NS2B/NS3	Polyprotein processing 40	A C-terminal fragment of NS2B and a protease domain of NS3 41
NS3	Polyprotein processing, viral replication and immune evasion 42	A protease domain and a helicase domain 43
NS4A	Membrane binding and homo-oligomerization 44	A N-terminal cytoplasmic region and a transmembrane segment (45
NS4B	Viral replication and immune evasion 46	Three transmembrane helices and two helices that peripherally associate with the membrane 47
NS4	Pathogenesis, viral replication, membrane binding and homo-oligomerization 48	A N-terminal cytosolic region and four predicted transmembrane segments (pTMSs) 49
NS5	Viral replication and immune evasion 50	A N-terminal methyltransferase (MTase) domain and a C-terminal RNA-dependent RNA polymerase (RdRp) 51

Notes: C is the abbreviation of capsid
